# Not Just a Sore Throat: A Case of a Retropharyngeal Abscess Causing Midline Shift of the Nasopharynx

**DOI:** 10.7759/cureus.21336

**Published:** 2022-01-17

**Authors:** Michelle Hernandez, Ariel Vera, Bryan Kwon, Latha Ganti

**Affiliations:** 1 Emergency Medicine, University of Central Florida College of Medicine, Orlando, USA; 2 Emergency Medicine, Envision Physician Services, Plantation, USA; 3 Emergency Medicine, Osceola Regional Medical Center, Kissimmee, USA; 4 Emergency Medicine, Hospital Corporation of America Healthcare Graduate Medical Education Consortium Emergency Medicine Residency Program of Greater Orlando, Orlando, USA; 5 Emergency Medicine, Brown University, Providence, USA

**Keywords:** suppurative cervical lymphadenitis, airway compromise, deep neck space infection, pharyngitis, retrophraryngeal abscess

## Abstract

A retropharyngeal abscess (RPA) is a deep neck space infection that can present with subtle symptoms. Although it is an uncommon diagnosis, an RPA can be life-threatening as it can result in airway compromise if not treated promptly. In this article, we report a case of a 21-month-old infant with a retropharyngeal abscess that required prompt recognition and treatment.

## Introduction

A retropharyngeal abscess (RPA) is an uncommon but potentially life-threatening diagnosis. It is defined as an abscess in the space between the posterior pharynx and the prevertebral fascia [1]. Although an infrequent diagnosis, the incidence of the retropharyngeal abscess has been increasing in recent years [1,2]. RPA is most commonly seen in boys under the age of five years and has a winter-spring seasonality [2]. Most cases occur with a reported antecedent upper respiratory infection (URI). An URI with suppurative cervical lymphadenitis can progress to a retropharyngeal abscess [1]. A retropharyngeal abscess is also seen as a complication after direct inoculation with a foreign body, such as fish bones, chicken bones, and pens [3]. Delay in the diagnosis and management may lead to potentially lethal complications [4].

## Case presentation

A 21-month-old female with no past medical history was brought in by her mother with a sore throat and fever. The patient was complaining of throat pain for the last two days, so the patient’s mother took her to the pediatrician. The patient had a positive rapid strep test and was prescribed amoxicillin and acetaminophen earlier that day. A few hours after leaving the pediatrician’s office, her mother noticed the patient was not turning her head to the left. The patient was refusing to eat food and was only drinking small sips of water. Her mother also reported episodes of mild drooling, which prompted evaluation at the emergency department (ER).

Upon arrival to the ER, the patient was febrile with a temperature of 39.4°C and tachycardic with a heart rate of 188 beats per minute. The patient’s airway was intact without stridor or drooling upon initial assessment. She was in the hospital bed laying still while her sibling was running around, which her mother stated was very atypical. Instead of turning her head, the patient would turn her entire body when asked to look in the other direction. The history of sore throat, fever, decreased oral intake with mild drooling, and torticollis was concerning for a deep space infection.

**Figure 1:**
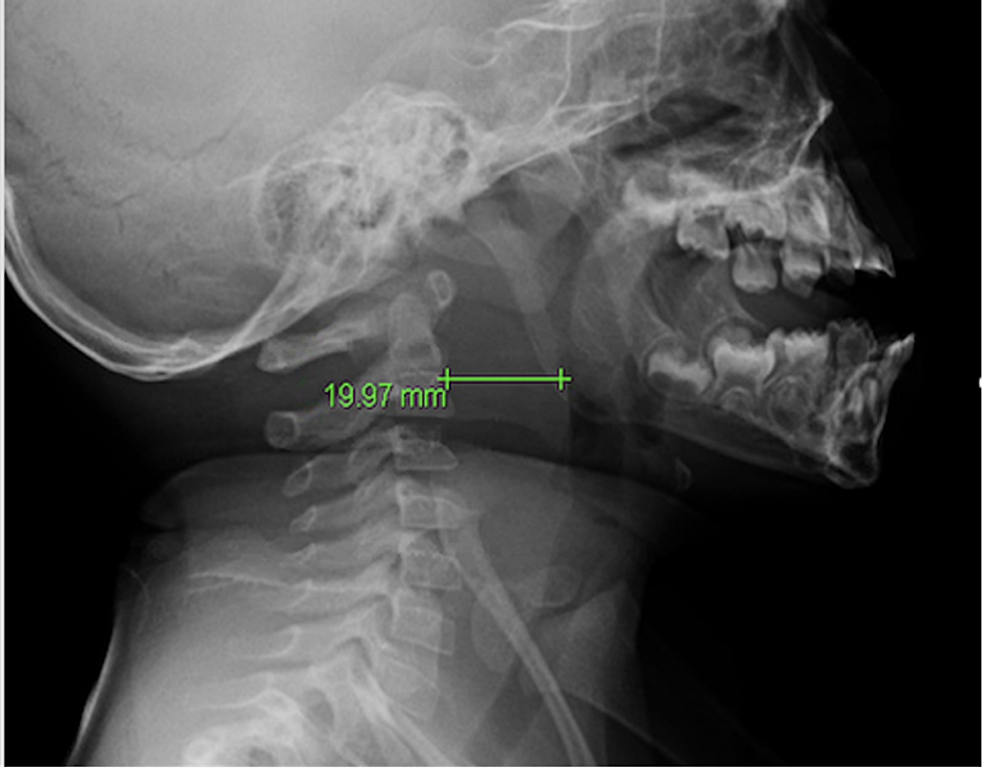
Radiograph of the neck shows significant soft tissue swelling indicating cellulitis or an abscess in the retropharyngeal space

Laboratory analyses revealed a leukocytosis of 22,000/uL with neutrophilic predominance. All other laboratory results including hematocrit, platelets, electrolytes, and creatinine were within normal limits. A radiograph of the neck and soft tissue revealed significant prevertebral soft tissue swelling suggestive of cellulitis or an abscess in the retropharyngeal space (Figure 1).

**Figure 2:**
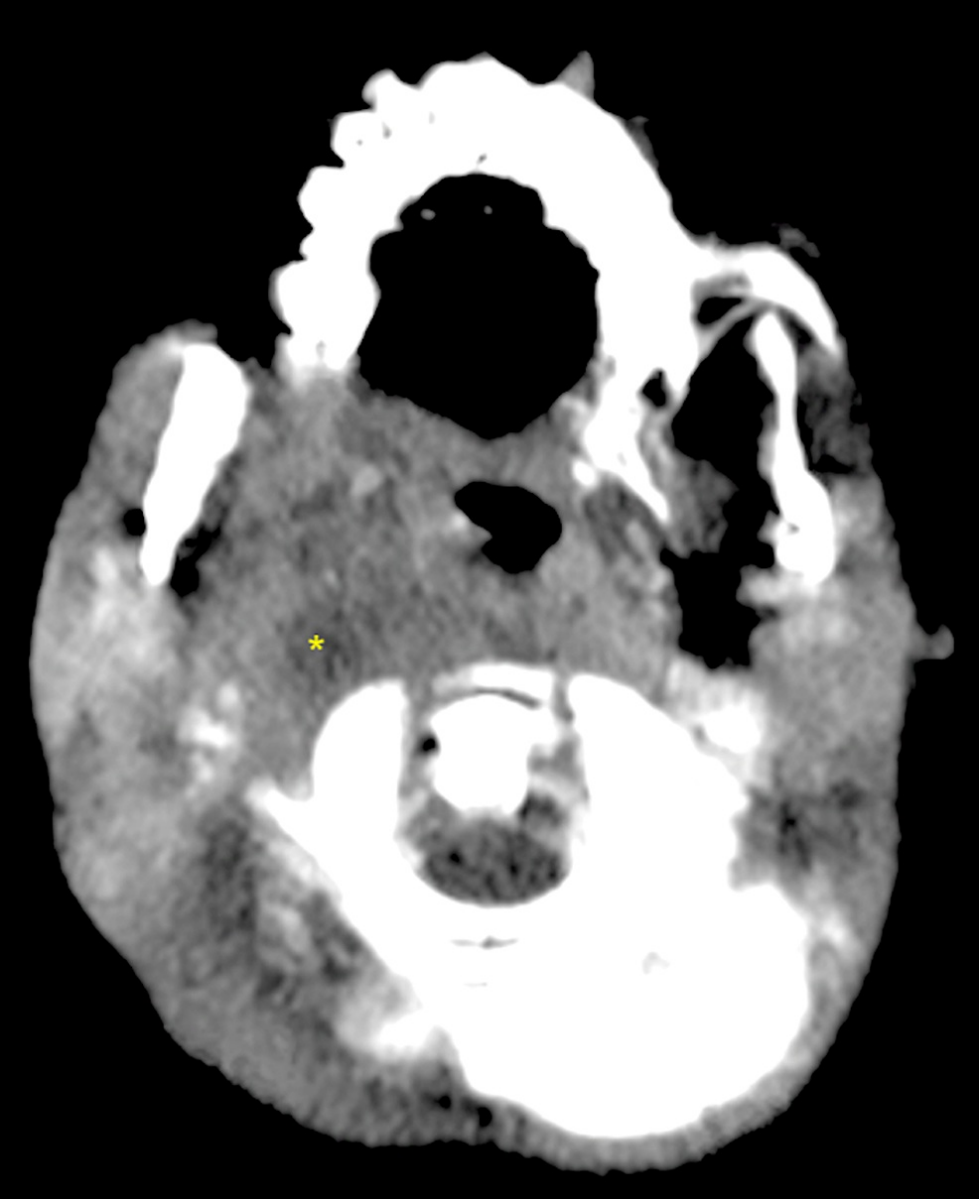
CT of the neck with contrast shows retropharyngeal abscess (2 cm x 2 cm x 2.5 cm) on the right with a mass effect on the nasopharynx (asterisk) CT, Computed tomography.

Computed tomography (CT) scan revealed a 2 cm x 2 cm x 2.5 cm retropharyngeal abscess with a mass effect on the airway (Figure 2).

Otolaryngology was consulted emergently. Our facility did not have a pediatric otolaryngologist, so arrangements were promptly made for transfer to the pediatric hospital nearby. The patient underwent surgery the next morning, and 1 cc of purulent material was removed. She had an uncomplicated post-surgical course and was discharged three days later with amoxicillin. The culture from the purulent specimen demonstrated group A *Streptococcus* growth.

## Discussion

Early presenting symptoms of RPA are usually indistinguishable from uncomplicated pharyngitis, so a detailed history and physical examination is a key to avoid delay in the diagnosis. Early symptoms include sore throat, fever, and torticollis [5]. Late symptoms include stridor, respiratory distress, drooling, and neck stiffness [5]. If there is a concern for deep space infection, a lateral radiograph of the neck can be taken to visualize the soft tissue. The radiograph of the neck should be taken at the end of inspiration for accuracy. The prevertebral space should be less than 7 mm at C2 and less than 14 mm at C6 in the pediatric population. In a stable patient, a CT scan of the neck with contrast is ideal for diagnosis.

In a study that assessed the accuracy of the CT scan for the diagnosis of deep neck infections in pediatrics, the overall accuracy was 63% [6]. Since the treatment of a retropharyngeal abscess ranges from prolonged courses of intravenous antibiotics to surgical incision and drainage, CT results combined with clinical findings should guide the decision of surgery [6]. Typically, surgery is indicated when the abscess is greater than 2 cm on the CT scan and when there are complications or worsening of symptoms [6]. Antibiotics should be administered as soon as possible; clindamycin, cefoxitin, or ampicillin/sulbactam provide adequate coverage. In patients who do not respond to initial treatment, vancomycin or linezolid should be started for potentially resistant gram-positive cocci [6]. Intravenous antibiotics should be administered continuously until the patient clinically improves and becomes afebrile [7]. Steroids can also be given to decrease soft tissue swelling.

It is important to note that a small amount of edema in the airway can result in significant obstruction in pediatric patients. Therefore, a retropharyngeal abscess should be treated as an airway emergency, and rapid assessment of the airway is the first step. Emergent ear, nose, and throat (ENT) consult should be obtained. If there is a concern for severe airway obstruction, an anesthesiologist and a critical care specialist should also be notified to assist [1].

## Conclusions

A retropharyngeal abscess is an uncommon but potentially life-threatening diagnosis. Initial symptoms may be subtle and indistinguishable from uncomplicated pharyngitis. If a clinician suspects an RPA, a CT scan of the neck with contrast should be obtained. Airway management is a top priority, and otorhinolaryngology should be consulted emergently. Clinical findings and imaging should be analyzed together to determine if incision and drainage in the operating room are necessary.
